# Prevalence of Cryptococcal Antigenemia and Lateral Flow Assay Accuracy in Severely Immunosuppressed AIDS Patients

**DOI:** 10.3390/jof10070490

**Published:** 2024-07-16

**Authors:** Adriana Carla Garcia Negri, Maína de Oliveira Nunes, Gláucia Moreira Espíndola Lima, James Venturini, Sandra Maria do Valle Leone de Oliveira, Márcia dos Santos Lazera, Lídia Raquel de Carvalho, Marilene Rodrigues Chang, Rosianne Assis de Sousa Tsujisaki, Adriana de Oliveira França, Rinaldo Poncio Mendes, Anamaria Mello Miranda Paniago

**Affiliations:** 1 Graduate Program in Infectious and Parasitic Diseases, Faculty of Medicine, Federal University of Mato Grosso do Sul, Campo Grande 79070-900, MS, Brazil; drikarla@gmail.com (A.C.G.N.); james.venturini@ufms.br (J.V.); sandra.leone@fiocruz.br (S.M.d.V.L.d.O.); marilene.chang@ufms.br (M.R.C.); dricaseal@gmail.com (A.d.O.F.); tietemendes@terra.com.br (R.P.M.); 2 Maria Aparecida Pedrossian University Hospital, Federal University of Mato Grosso do Sul, Campo Grande 79080-190, MS, Brazil; mainanunes@gmail.com (M.d.O.N.); glauciaespindola18@gmail.com (G.M.E.L.); 3 Oswaldo Cruz Foundation, Campo Grande 79081-746, MS, Brazil; 4 National Institute of Infectious Diseases Evandro Chagas, Oswaldo Cruz Foundation, Rio de Janeiro 21040-900, RJ, Brazil; 5 Department of Biostatistics, Plant Biology, Parasitology and Zoology, Bioscience Institute, São Paulo State University, Campus de Botucatu, Botucatu 18618-687, SP, Brazil; lidia.carvalho@unesp.br; 6 Faculty of Pharmaceutical Sciences, Food and Nutrition, Federal University of Mato Grosso do Sul, Campo Grande 79070-900, MS, Brazil; 7 Department of Nutrition, Health Sciences Center, UFB—Universidade Federal da Paraíba, João Pessoa 58091-900, PB, Brazil; 8 Department of Tropical Diseases, Botucatu Medical School, São Paulo State University, Botucatu 18618-687, SP, Brazil

**Keywords:** cryptococcosis, HIV infection, immunocromatography, point-of-care systems

## Abstract

This study aimed to estimate the prevalence of cryptococcal antigenemia detected by lateral flow assay (LFA) in AIDS patients and its accuracy in the diagnosis of cryptococcosis. Conducted at a university hospital in Brazil from March 2015 to July 2017, it included AIDS patients over 18 years old with a CD4+ count ≤ 200 cells/mm^3^. Cryptococcal antigen (CrAg) detection using LFA and latex agglutination (LA), along with blood and urine cultures, were performed. The reference standard was the identification of *Cryptococcus* spp. in clinical specimens through microbiological or histopathological examination. Among 230 patients, the prevalence of CrAg detected by LFA (CrAg LFA) was 13.0%. Factors associated with cryptococcal antigenemia included fever, vomiting, seizures, and a lack of antiretroviral therapy. The sensitivity and specificity of CrAg LFA were 83.9% and 98.0%, respectively. The positive predictive value (PPV) was 86.7%, the negative predictive value (NPV) was 97.5%, and overall accuracy was 96.1%. Cross-reactions were observed in patients with histoplasmosis and paracoccidioidmycosis, but not with aspergillosis or positive rheumatoid factor. The study concludes that the LFA is a useful tool for detecting cryptococcal antigenemia in severely immunocompromised AIDS patients due to its high NPV, specificity, and PPV.

## 1. Introduction

Cryptococcosis remains one of the most frequent and life-threatening opportunistic infections in people with AIDS. It is estimated that cryptococcal meningitis (CM) causes 19% of the AIDS-related deaths globally [1]. In cryptococcosis, the fatality rates have been reported in 45 to 65%. This is partly due to the delay in diagnosis, which in the case of cryptococcosis requires clinical suspicion and access to complex procedures, such as cerebrospinal fluid (CSF) puncture in hospital settings. However, detection of cryptococcal antigen (CrAg) in serum has been shown to be an accurate method for diagnosis, able to be detected 22 days (range 5–234) before meningitis symptoms appear [2], making it possible for antigenemia to predict meningitis in advanced stages of HIV infection [3].

In addition to being useful for the diagnosis of active disease, studies show that preemptive treatment of cases with only asymptomatic antigenemia can reduce the incidence of CM [4].

CrAg can be detected by different methods, such as LA reaction, enzyme immunoassay, and immunochromatography (Lateral Flow Assay-LFA). The latter is regarded as a point-of-care exam because it can be easily and rapidly performed, with results available in 15 min. It is particularly useful both for quick decision-making in the emergency room and for screening of asymptomatic patients, considering that among these, about 20% never come back for a follow-up appointment [5,6].

The World Health Organization (WHO) has recommended LFA—a strategy shown to be cost-effective [7]—for detecting CrAg as screening in AIDS patients with low CD4+ cell count in regions with a prevalence of cryptococcosis greater than 3%.

Although several studies have been performed on CrAg-LFA, an evaluation of cross reactions with different systemic mycoses, including paracoccidioidomycosis (PCM), which is widely spread in Latin America, and of its accuracy in severely immunosuppressed AIDS patients, must be carried out.

The aim of the present study was to estimate the prevalence and the determinants of cryptococcal antigenemia using LFA in severely immunosuppressed AIDS patients, as well as to analyze its accuracy and cross reactions in cryptococcosis diagnosis.

## 2. Patients and Methods

### 2.1. Study Design and Setting

A prospective data collection study was conducted in the Reference Service for Infectious and Parasitic Diseases at the University Hospital Maria Aparecida Pedrossian (UHMAP), Federal University of Mato Grosso do Sul—UFMS Campo Grande (MS)—School of Medicine, Brazil, from March 2015 to July 2017. Consecutive AIDS patients older than 18 years old with CD4+ count ≤ 200 cells/mm^3^, with or without any clinical manifestations, were eligible. They were invited to participate in the study at the time of blood collection for HIV viral load and CD4+ cell count determinations, which had been ordered by their attending physician. Patients who had previously had cryptococcosis were excluded.

In addition to CrAg LFA prevalence and its risk factors, the accuracy of CrAg LFA was analyzed, and results of the different diagnostic tests were compared.

### 2.2. Sample Size

The lowest number of patients to be evaluated (N) was calculated using the following formula:

$$N=\frac{{\left[z_{\alpha }.\sqrt{\psi +1}+z_{\beta }.\sqrt{(\psi +1)-p.{\left(\psi -1\right)}^{2}}\right]}^{2}}{p.{\left(\psi -1\right)}^{2}}$$

The sample size to assess the difference between two proportions in the same individuals using the McNemar’s test as the basis was 230 participants. It was calculated according to Zar [8] specifications, considering an alpha error equal to 0.05 with a 90% test power and a ratio between the discordant findings of 2.2.

### 2.3. Study Procedures

Data collection. Clinical, sociodemographic, and laboratory variables were prospectively recorded on a standardized form. Clinical progress data, including deaths, were obtained from medical records.

Biological samples. Peripheral blood and urine samples were collected at the time of allocation in the study. Sera from patients of the group with other diseases were obtained from the biorepositories of São Paulo State University—UNESP Botucatu, School of Medicine, Brazil (histoplasmosis and aspergillosis) and Federal University of Mato Grosso do Sul—UFMS Campo Grande—Faculty of Medicine, Brazil (paracoccidioidomycosis). Sera from patients with rheumatoid disease and positive rheumatoid factor were collected at the Rheumatology Clinic of the UHMAP.

### 2.4. Diagnostic Tests

The patients were submitted to the procedures summarized below, and all tests were performed with samples collected when patients were allocated to the study.

(1) Lateral flow assay (LFA) was performed in serum using the CrAg LFA kit from Immuno-Mycologics, Inc. (Norman, OK, USA), according to the manufacturer’s instructions. All tests were carried out just after blood was drawn and were read by the same trained person in the study, who was blinded to the other tests.

Samples from healthy subjects had been stored at −20 °C for six years before they were tested.

(2) Latex agglutination (LA). The serum samples obtained at admission to the study were kept in a freezer at −80 °C until the moment of their evaluation. All of them were tested using the same latex kit (CrAg CALAS—Meridian Biosciences, Inc., Cincinnati, OH, USA) and, at the same time, according to the manufacturers’ instructions. This test was performed by a single professional who was unaware of the patient’s symptoms and results of other tests.

(3) Blood culture was performed using two methods: (a) the automated method (BD BACTEC™) and (b) the conventional method with seeding on Sabouraud Dextrose Agar (SDA) with chloramphenicol.

(4) Urine culture was performed by the conventional method. A 20–40 mL midstream clean-catch urine sample was collected by the own patient in the morning. For women, the periurethral area and perineum were appropriately cleaned beforehand. All the urine specimens were transported promptly to the laboratory and processed within two hours after collection. SDA with chloramphenicol was used as culture medium.

(5) Fungal identification. After observing the growth of yeasts on SDA, the culture was checked for purity (absence of bacteria) by microscopy. Subsequently, the isolates were incubated in automated identification equipment (BD Phoenix™, Becton Dickinson, Franklin Lakes, NJ, USA) and subcultured on Niger seed agar plates. Differentiation between the species of *Cryptococcus neoformans* complex and the species of *Cryptococcus gattii* complex was carried out using L-canavanine-glycine-bromothymol blue (CGB) medium [9].

(6) Molecular typing of *Cryptococcus* spp. was performed using the Polymerase Chain Reaction-Restriction Fragment Length Polymorphism (PCR-RFLP) technique according to Meyer et al. (2003) [10].

DNA extraction was performed with phenol:chloroform:isoamyl alcohol 25:24:1 (*v*/*v/v*) (Sigma, Saint Louis, MI, USA), according to the Latin American Cryptococcal Network Project protocol modified from Ferrer et al. (2001) [11]. The URA5 gene (≈750 bp) was amplified by PCR using primers URA5 (5′ ATGTCCTCCCAAGCCCTCGACTCCG 3′) and SJ01 (5′ TTAAGACCTCTGAACACCGTACTC 3′) (Sigma). The amplified products were digested with 20,000 U/mL HhaI (New England Biolabs, Ipswich, MA, USA) and 5000 U/mL Sau96I (New England Biolabs, Ipswich, MA, USA), and separated by electrophoresis on 2% agarose gel.

The obtained RFLP profiles were analyzed by visual comparison with band-profile strain references: C. neoformans WM 148 (serotype A, VNI), WM 626 (serotype A, VNII), WM 628 (serotype AD, VNIII), WM 629 (serotype D, VNIV); and C. gattii WM 179 (serotype B, VGI), WM 178 (serotype B, VGII), WM 175 (serotype B, VGIII), WM 779 (serotype C, VGIV) [10,12].

Even in cases in which CrAg LFA, blood culture, or urine culture was positive, or in patients with clinical manifestations suggestive of cryptococcosis, the following tests were performed in CSF and/or in other biological samples:

(a) Mycological examination with India ink staining.

(b) Fungal culture and identification by the conventional methods as described above.

(c) Histopathological examination of biopsied or surgically excised tissue, with hematoxylin & eosin staining and/or, when necessary, Gomori-Grocott staining.

### 2.5. Medical Evaluation, Treatment, and Follow-Up

The result of the LFA test was delivered to the patient on the same day, and when it was positive, the patient was seen by the service physician. A lumbar puncture, preceded by cranial tomography, was performed if there was no contraindication in order to rule out CM. Other tests were requested, depending on the indication of the attending physician.

The treatment of cryptococcosis in the service follows the Infectious Diseases Society of America guidelines. An alternative regimen was used in the induction therapy (amphotericin B plus fluconazole) because flucytosine was not available. For patients with only asymptomatic antigenemia, preemptive treatment with fluconazole was prescribed [13].

Data on occurrence and cause of death up to five years of follow-up were obtained from the medical record.

### 2.6. Statistical Analysis

The association analysis to positive LFA, in comparing categorical variables for independent samples, was performed by a chi-square test, followed by the Goodman test when indicated.

Confounding effects were minimized by performing binary logistic regression, adjusted for potential confounders identified in the analysis. Such potential confounders are variables found to have *p* ≤ 0.20.

For CrAg LFA (index test) accuracy analyses, the diagnosis performed by identification of *Cryptococcus* spp. at any clinical specimen by mycological evaluation—either direct examination or culture—or histopathological examination was used as a reference standard.

Sensitivity, specificity, positive predictive values (PPV) and negative predictive values (NPV), and positive and negative likelihood ratios were calculated as to previous specifications [14].

McNemar’s test was used to compare every two diagnostic methods performed in the same sample.

The survival curves were constructed regarding cryptococcosis comorbidity, place of patient’s assistance and/or admission, gender, age group, and CD4+ cell count using the Kaplan-Meier survival curves, compared by the log-rank test.

Significance was set up at *p* ≤ 0.05. Statistical analyses were performed using SAS—Statistical Analysis System version 6.12, SAS Institute, Inc., Cary, NC, USA.

### 2.7. Ethical Considerations

The Ethics Committee of the Federal University of Mato Grosso do Sul approved the present study (number 912.054, 11 December 2014). All patients signed a written statement of informed consent for participation.

Preliminary results of this study were presented at the *Cryptococcus* Network Symposium in the 10th International Conference on *Cryptococcus* and Cryptococcosis, Foz do Iguaçu, Paraná state (Brazil), 2017

## 3. Results

**Figure 1:**
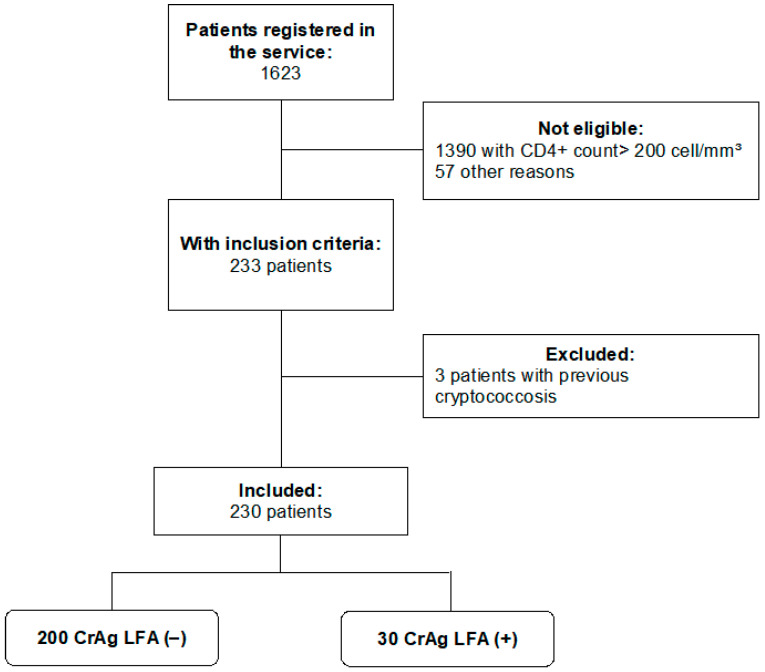
Flowchart of participants through the study. Abbreviations: CrAg LFA: Antigen *Cryptococcus* lateral flow assay.

Two hundred and thirty patients were included in the study (Figure 1), most of them men (73.9%). The age was 41.2 ± 11.4 years old, and the CD4+ count was 83.4 ± 61.7 cells/mm^3^. They had an average of 4.7 ± 5.0 years of HIV diagnosis, and 160 of them had at least one opportunistic disease, such as neurotoxoplasmosis (n = 61); visceral leishmaniasis (n = 49); tuberculosis (n = 35); cytomegaloviruses (n = 26); and histoplasmosis (n = 8).

The prevalence of cryptococcal antigenemia determined by CrAg LFA was 13.0% (95% CI, 9.3–18.0).

Fever, vomiting, seizures, or not having started antiretroviral therapy (ART) were variables independently associated with positive CrAg LFA (Table 1).

**Figure 2:**
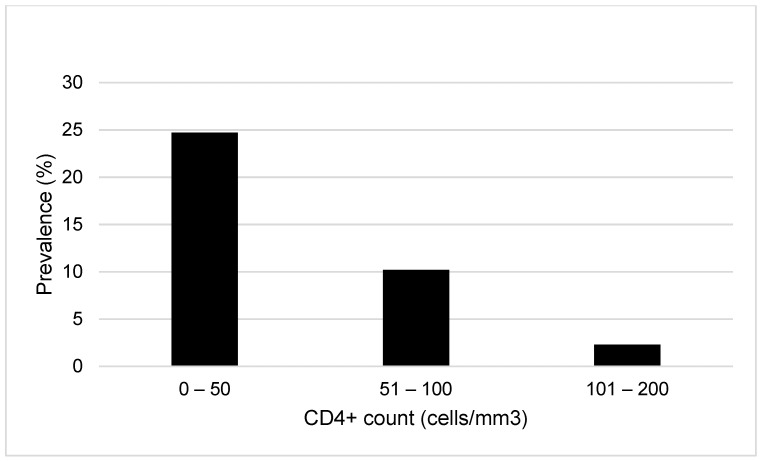
Prevalence of cryptococcal antigenemia determined by lateral flow assay according to CD4+ cell count.

The higher prevalence of antigenemia was observed in patients with up to 50 CD4+ cell/mm^3^, in those with neurological symptoms, and in the ART-naïve ones (Figure 2, Table 1).

Among the 30 CrAg LFA-positive patients, the most frequent symptoms were fever and headache. Table 2 shows the symptoms regarding CrAg LFA results.

Fever, vomiting, seizures, and antiretroviral therapy (ART)-naïve were variables independently associated with CrAg LFA positivity (Table 3).

In the tests comparing every two results, it is observed that LFA had remarkably similar results to LA, to culture, and to direct examination of *Cryptococcus* in the CSF (*p* > 0.05) (Table 4). Agreements between each pair of tests are shown in Table S1.

Cryptococcosis was confirmed in the 29 patients by at least one of the following parameters: histopathological examination in 4 (13.8%), direct mycological examination in 19 (65.5%), and culture in 26 (89.7%) of them. Culture in CGB medium demonstrated that all of them belonged to the *Cryptococcus neoformans* complex. However, only 14 of these 26 fungi were genotyped—12 (85.7%) were identified as VNI and 2 (14.3%) as VNII molecular types (nowadays, both are called *Cryptococcus neoformans*).

Taking into account a control group constituted by 100 healthy blood donors, the LFA showed very good accuracy parameters (Table 5).

**Figure 3:**
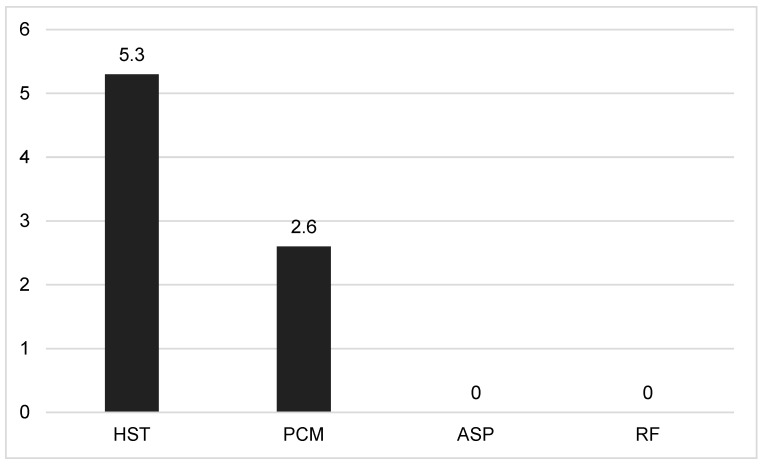
Proportion of CrAg LFA cross-reactions in paracoccidioidomycosis (PCM) (n = 38), histoplasmosis (HIST) (n = 19), aspergillosis (ASP) (n = 22) sera, and rheumatoid disease patients with positive rheumatoid factor (RF) (n = 23).

Cross reactions were observed in serum samples from patients with PCM (2.6%) and histoplasmosis (5.3%), which did not differ (*p* = 0.89); however, for patients with aspergillosis or rheumatoid disease with positive rheumatoid factor, no cross reactions were found (Figure 3).

**Figure 4:**
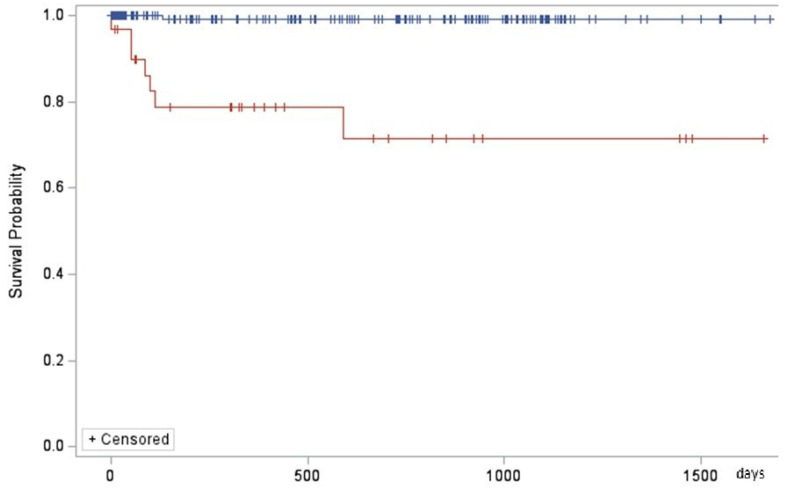
Kaplan-Meier estimator related to presence of cryptococcosis (blue line: absence, red line: presence) (*p* < 0.001).

During the follow-up of the 230 patients, 41 of them died—8 due to cryptococcosis and 33 due to other causes. The analysis of the survival curves in five years showed that the survival time was shorter in patients with cryptococcosis (Figure 4) and in those who were hospitalized, while sex, age group, and CD4+ cell count presented no effect (Figures S1–S4).

## 4. Discussion

The gold standard for cryptococcosis diagnosis remains the fungal identification in mycological examination and isolation in CSF culture and other clinical samples. However, LFA detection of serum antigens has been widely used, especially in screening programs for this mycosis, as it is easy to be performed, it does not need laboratory facilities for such, and its results are available in a few minutes [3,7]. The present study estimated the prevalence of cryptococcal antigenemia by LFA in 230 consecutive adult patients with AIDS and CD4+ count ≤ 200 cells/mm^3^ without previous diagnosis of cryptococcosis, analyzed its accuracy parameters, and compared its sensitivity with that of other diagnostic tests.

The cryptococcal antigenemia prevalence rate found was 13.0% (95% confidence interval (CI) 9.3–18.0). The prevalence of cryptococcal antigenemia worldwide has ranged from 1.2 to 21.0%, depending on the geographic localization, age, clinical meningitis presence, and CD4+ cell count of the patients included in the study [15,16,17,18,19,20]. In countries with an incidence rate of cryptococcosis, such as Ethiopia, Congo, and Kenya, the LFA positivity rate in the screening program was 15.2%, 13.4%, and 13.7% [21], respectively. On the other hand, in countries like Germany with low cryptococcosis incidence, the LFA positivity rate was 1.2% [20].

Children were not included in the present study. Cryptococcosis occurs in both children and adolescents, yet in lower frequency than in adults [22]. A study carried out in South Africa showed that LFA positivity increases as to age groups: 1% in children up to 1 year old, 3.7% in patients up to 19 years old, and 5.8% in those over 19 years old [22].

A Brazilian study revealed a prevalence of 3.1% (95% CI, 1.0–7.0) in adult HIV-infected inpatients with <200 CD4+ cells/μL, but those with symptomatic meningitis were excluded [16]. A number of studies on the prevalence of cryptococcal antigenemia included only asymptomatic patients; however, when symptomatic patients were included, the prevalence rate could reach 13.2% to 21.0%, as seen in investigations conducted in Madagascar and Cambodia, respectively [23,24].

In the present study, the LFA positivity rate was higher in AIDS patients with CD4+ count ≤ 100 cells/mm^3^, with a predominance in those with ≤50 cells/mm^3^, but two cases (4.2%) with 101 to 200 cells/mm3 were identified. A systematic review that meta-analyzed 21 studies comparing antigenemia prevalence at CD4+ count ≤ 100 cells/μL vs. 101–200 cells/μL reveals an odds ratio of 2.5 (95% CI, 1.9–3.3) [25]. The WHO has recommended screening of cryptococcal antigenemia for ART-naïve patients with CD4+ count ≤ 100 cells/mm^3^, followed by preemptive antifungal therapy for the positive ones, as this strategy has been proven to be cost-effective [26,27,28]. In the same guidelines, the screening for patients with 101–200 cells/mm^3^ was suggested, despite being less cost-effective [26,27]. It is important to emphasize that CD4+ count ≤ 200 cells/mm^3^ is a factor strongly associated with the risk of AIDS mortality [29].

In the present study, ART-naïve patients were more likely to have positive CrAg LFA. Nevertheless, a systematic review shows no difference between studies that recruited only ART-näive patients (32 studies with 18,657 patients) and those that included both ART-naïve and ART-experienced patients (16 studies with 6950 patients) [25].

Fever, vomiting, and seizure were factors independently associated with cryptococcal antigenemia by LFA. While seizure is a very suggestive manifestation of neuroinfection in AIDS patients, fever and vomiting are common in other AIDS opportunistic diseases. Fever presented low PPV but high NPV for cryptococcosis in AIDS patients in a study carried out in Ethiopia [18]. Thus, a positive CrAg LFA test in the serum of patients with these symptoms can help to screen this diagnosis.

More than a quarter (28.3%–65/230) of the patients allocated in this study underwent lumbar puncture, so they had a strong clinical suspicion of neuroinfection, which may have contributed to the high prevalence rate found. CM was the most frequent neuroinfection among them, followed by neurotoxoplasmosis.

Comparison between LFA and LA showed concordant results, but in 31 patients they were discordant. Both tests detect antigenemia; however, while LA demands a laboratory structure, LFA is easier and faster to perform, and it can be considered a point-of-care test. A study with 634 participants comparing LA and LFA showed excellent agreement between the tests [30].

Cryptococcuria detection has been useful in CM and disseminated cryptococcosis [31,32,33]. In this study, serum LFA showed higher positivity than urine culture for cryptococcosis diagnosis, but Staib’s medium was not used as it has been shown to be more selective [33].

All the 14 genotyped clinical samples belonged to the C. neoformans complex, 12 of them VNI and 2 VNII, with higher VNI prevalence, confirming previous reports for AIDS patients [12].

LFA sensitivity of 83.9% was not so high as in other studies—99.3% [34] and 100.0% [35]. Nonetheless, the NPV of 97.5% was so high as another study [34].

Microbiological tests in cryptococcosis can be false negative, especially in the early stages of CM [34]. On the other hand, capsular antigens of the fungus can be detected in the serum up to 20 days before its visualization in the CSF by direct examination with India ink stain or its growth in culture [2]. A systematic review with meta-analysis found pooled sensitivity of 97.6% (95% CI, 95.6–98.9%) and pooled specificity of 98.1% (95% CI, 97.4–98.6%) for LFA in serum specimen from 3407 inpatients suspected with cryptococcosis. Even though high heterogeneity had been detected among the studies [36], such values were higher than the ones in the present study.

The comparison of positivity among diagnostic tests revealed that serum CrAg LFA was higher than blood and urine cultures, tended to be higher than CSF direct examination stained with India ink, and did not differ from LAT-CrAg in serum or CSF culture.

The comparison of positivity among diagnostic tests revealed that serum CrAg LFA was higher than blood and urine cultures, tended to be higher than CSF direct examination stained with India ink, and did not differ from LA-CrAg in serum or CSF culture.

Cross-reactions of CrAg LFA were observed with serum from patients with confirmed histoplasmosis (5.3%) and PCM (2.6%). Histoplasmosis is a frequent opportunistic disease in AIDS patients, and this cross-reaction may be a problem, in spite of the differences in clinical picture, including in AIDS patients. In addition, the confirmation of histoplasmosis is usually difficult in countries where methods for the identification of antigens are not available.

Paracoccidioidomycosis is an endemic condition in Latin America, found mainly in the region where the present study was performed [37]. It is uncommon in AIDS patients; whereas cases have been reported [38], only 1.4% of deaths caused by fungal diseases in Brazilian AIDS patients were attributed to PCM [39]. Its mycological diagnosis is easier than that of histoplasmosis because of its typical yeast forms. The involvement of the central nervous system (CNS), so common in cryptococcosis, has been observed in few cases of PCM [40]. Thus, PCM as a differential diagnosis should always be investigated in endemic regions, since its treatment and control of cure are completely unlike those of cryptococcosis.

Differently from the present study, cross-reactions in CrAg LFA have not been found, but cross-reactions in CrAg LA have been reported with blastomycosis, aspergillosis [41], and *Trichosporon asahii* infections [42]. Nevertheless, the most known cause of cross reactions was rheumatoid factors [41]; nowadays, treatment of serum specimens with pronase removes rheumatoid factor interference [43].

The lower survival rate of AIDS patients with cryptococcosis co-morbidity in relation to other opportunistic diseases is well known, and it is due to the high case-fatality rate of this mycosis with a 55% rate among patients from low- and middle-income countries [44]. In the same service of the present study, the case fatality rate has been reported as 45% [45].

The severity of the disease justifies screening strategies with CrAg LFA in serum for early diagnosis with subsequent preemptive treatment for cases whose only evidence of cryptococcosis is just positive antigenemia. CrAg LFA in serum followed by preemptive antifungal therapy with fluconazole (screening and treatment strategy) [26] reduces CM mortality, particularly in severely immunosuppressed HIV patients [46].

Limitations included the sample at a single center and use of stocked specimens in groups with other diseases.

In conclusion, the high prevalence of antigenemia and cryptococcosis detected by CrAg LFA in the present study, performed by actively searching among severely immunocompromised AIDS patients, was alike that observed in other developing countries. Therefore, this finding justifies the implementation of the screening and preemptive treatment strategy, as recommended in the WHO guidelines. LFA is easy to perform, and its results are available in the same appointment, enabling an early decision about the patient. In addition, its high NPV, specificity, and PPV reinforce its routine use in severely immunosuppressed AIDS patients. However, cross reactions with histoplasmosis and PCM deserve special attention and require further investigations.

## Figures and Tables

**Table 1:** Evaluation of 230 AIDS patients with CD4+ count ≤ 200 cells/mm^3^. Prevalence of cryptococcal antigenemia determined by lateral flow assay (CrAg LFA) according to clinical and demographic variables. Chi-square test or Fisher’s exact test.

Variables	CrAg LFA Positive/Tested (%)	*p* Value
Gender		0.21
Male	25/170 (14.7)	
Female	5/60 (8.3)	
Age group (years old)		0.28
≤29	3/32 (9.4)	
30–39	15/79(19.0)	
40–49	6/69 (8.7)	
50–59	6/37 (16.2)	
60–69	-/7 (0.0)	
≥70	-/6 (0.0)	
CD4+ count (cells/mm^3^)		<0.0001 *
0–50	23/93 (24.7) A	
51–100	5/49 (10.2) B	
101–150	-/43 (0.0) B	
151–200	2/45 (4.4) B	
Neurological symptoms		<0.0001
No	-/63 (0.0)	
Yes	30/167 (19.0)	
HIV viral load (copies/μL)		0.08
Negative (<40)	3/51 (5.9)	
Positive (≥40)	27/179 (15.1)	
HIV diagnosis time (years)		0.42
0–5	18/144 (10.7)	
6–10	5/50 (10.0)	
>10	7/36 (19.4)	
ART-naïve		<0.0001
No	22/209 (10.5)	
Yes	8/21 (38.1)	

ART: antiretroviral therapy; * Goodman test. Values followed by the same letter are not significantly different (*p* > 0.05), and values followed by different letters present significant differences (*p* ≤ 0.05).

**Table 2:** Distribution of 230 AIDS patients with CD4+ count ≤ 200 cells/mm^3^ according to cryptococcal antigenemia by lateral flow assay (LFA) results, and neurological and general clinical manifestations.

Variables	LFA		*p* Value
	Positive (%)	Negative (%)	
	n = 30	n = 200	
Fever	21 (70.0)	46 (23.0)	<0.0001
Weight loss	8 (26.7)	44 (22.0)	0.57
Cough	6 (20.0)	18 (9.0)	0.07
Asthenia	1 (3.3)	45 (22.5)	0.01
Skin lesions	2 (6.7)	12 (6.0)	0.89
Vomiting	11 (36.7)	16 (8.0)	<0.0001
Diarrhea	4 (13.3)	24 (12.0)	0.83
Dyspnea	5 (16.7)	18 (9.0)	0.19
Abdominal pain	1 (3.3)	12 (6.0)	0.55
Nausea	6 (20.0)	16 (8.0)	0.04
Headache	28 (93.3)	98 (49.0)	<0.0001
Seizures	11 (36.6)	26 (13.0)	0.001
Somnolence	10 (33.3)	31 (15.5)	0.02
Mental confusion	9 (30.0)	28 (14.0)	0.03
Hemiparesis	4 (13.3)	34 (17.0)	0.61
Paraparesis	4 (13.3)	20 (10.0)	0.58
Miccional dysfunction	3 (10.0)	7 (3.5)	0.10
Erectile dysfunction	1 (3.3)	8 (4.0)	0.86
Paresthesia	2 (6.6)	20 (10.0)	0.56
Dysesthesia	3 (10.0)	6 (3.0)	0.06
Visual loss	11 (36.6)	57 (28.5)	0.36
Hearing loss	5 (16.6)	15 (7.5)	0.10
Memory deficit	4 (13.3)	34 (17.0)	0.61
Dysarthria	1 (3.3)	18 (9.0)	0.29
Disorientation	6 (20.0)	2 (1.0)	<0.0001

**Table 3:** Results of univariate and multivariate logistic regression analyses of factors associated with CrAg LFA positivity. Variables selected from Table 1 and Table 2, with univariate analysis showing *p* value up to 0.20.

Variables	LFA		*p*	COR (95% CI)	AOR (95% CI)
	Positive (%)	Negative (%)			
	n = 30	n = 200			
CD4+ count (cells/mm^3^)			<0.0001		
0–100	28 (93.3%)	114 (57.0%)		10.56 (2.45–45.55)	3.13 (0.32–30.36)
101–200	2 (6.7)	86 (43.0)		1	
HIV viral load (copies/μL)			0.09		
Positive (>40)	24 (80.0)	136 (68.0)		2.82 (0.81–9.80)	1.15 (0.24–5.42)
Negative (≤39)	3 (10.0)	48 (24.0)		1	
ART naïve			<0.0001		
Yes	8 (26.7)	13 (6.5)		5.23 (1.95–14.01)	6.11 (1.88–19.8)
No	22 (73.3)	187 (93.5)		1	
Symptoms					
Fever	21 (70.0)	46 (23.0)	<0.0001	7.81(3.34–18.23)	7.63 (2.96–19.71)
Cough	6 (20.0)	18 (9.0)	0.07	2.53 (0.91–6.99)	0.37 (0.06–2.36)
Asthenia	1 (3.3)	45 (22.5)	0.01	0.12 (0.02–0.89)	0.03 (0.0–0.6)
Vomiting	11 (36.7)	16 (8.0)	<0.0001	6.66 (2.70–16.40)	7.51 (2.58–21.86)
Dyspnea	5 (16.7)	18 (9.0)	0.19	2.02 (0.69–5.93)	0.52 (0.08–3.24)
Nausea	6 (20.0)	16 (8.0)	0.04	2.87 (1.03–8.05)	1.10 (0.15–8.06)
Headache	28 (93.3)	98 (49.0)	<0.0001	14.57 (3.38–62.81)	0.31 (0.04–2.11)
Seizures	11 (36.6)	26 (13.0)	0.001	3.87 (1.66–9.06)	3.01 (1.08–8.35)
Somnolence	10 (33.3)	31 (15.5)	0.02	2.72 (1.16–6.38)	2.00 (0.35–11.24)
Mental confusion	9 (30.0)	28 (14.0)	0.03	2.63 (1.09–6.33)	1.80 (0.48–3.9)
Miccional dysfunction	3 (10.0)	7 (3.5)	0.10	3.06 (0.75–12.56)	0.97 (0.06–14.36)
Dysesthesia	3 (10.0)	6 (3.0)	0.06	3.59 (0.85–15.21)	0.12 (0.01–1.37)
Hearingloss	5 (16.6)	15 (7.5)	0.10	2.47 (0.82–7.37)	0.71 (0.08–6.30)
Disorientation	6 (20.0)	2 (1.0)	<0.0001	24.75 (4.73–129.57)	2.50 (0.60–5.09)

ART: antiretroviral therapy; COR: crude odds ratio; AOR: adjusted odds ratio; CI: confidence interval.

**Table 4:** Pairwise comparison of positivity rates of various cryptococcosis diagnostic tests among 230 AIDS patients with CD4+ counts ≤ 200 cells/mm^3^ using the McNemar test.

Test 1 vs. Test 2	n	Test 1 Positivity n (%)	Test 2 Positivity n (%)	*p*
LFA vs. blood culture	221	29 (13.1)	16 (7.2)	0.002
LFA vs. LA	224	30 (13.4)	25 (11.2)	0.47
LFA vs. urine culture	226	30 (13.3)	11 (4.9)	<0.0001
LFA vs. CSF culture	65	27 (41.5)	21 (32.3)	0.18
LFA vs. CSF direct examination	64	26 (40.6)	18 (28.1)	0.06
Blood culture vs. LA	217	16 (7.4)	25 (11.5)	0.072
Blood culture vs. urine culture	217	16 (6.9)	11 (5.1)	0.096
Blood culture vs. CSF culture	64	16 (25.0)	20 (31.3)	0.285
Urine culture vs. CSF culture	65	11 (16.9)	21 (32.3)	0.025

LFA: lateral flow assay; LA: latex agglutination; CSF: cerebrospinal fluid; n: number of patients evaluated in each comparison.

**Table 5:** Accuracy parameters of the lateral flow assay for cryptococcosis diagnosis in AIDS patients with up to 200 CD4+ cells/mm^3^.

Parameters of Accuracy	Value (95% CI)
Sensitivity	83.9% (63.6%–94.6%)
Specificity	98.0% (94.9%–99.5%)
Positive predictive value	86.7% (70.9%–94.6%)
Negative predictive value	97.5% (94.6%–98.9%)
Positive likelihood ratio	41.7 (15.6–111.4)
Negative likelihood ratio	0.16 (0.07–0.37)
Accuracy	96.1% (92.7%–98.2%)

## Data Availability

Data are contained within the article and Supplementary Materials.

## Supplementary Materials

The following supporting information can be downloaded at: https://www.mdpi.com/article/10.3390/jof10070490/s1, Table S1. Agreement of various cryptococcosis diagnostic tests in pairs among 230 AIDS patients with CD4+ counts ≤ 200 cells/mm^3^; Figure S1. Kaplan-Meier estimator related to place of admission; Figure S2. Kaplan-Meier estimator related to sex; Figure S3. Kaplan-Meier estimator related to age group; Figure S4. Kaplan-Meier estimator related CD4+ cell count.
