# Supplementary material for: Prevalence of Cryptococcal Antigenemia and Lateral Flow Assay Accuracy in Severely Immunosuppressed AIDS Patients
Source: J Fungi (Basel). 2024 Jul 16;10(7):490. doi: 10.3390/jof10070490 (PMC11278224; doi:10.3390/jof10070490)
Supplement: Supplementary file 1 [file jof-10-00490-s001.zip › jof-3045790-supplementary.pdf]

Supplementary materials

Table S1. Agreement of various cryptococcosis diagnostic tests in pairs among 230 AIDS patients with CD4+ counts ≤ 200 cells/mm^3

Test A *vs* Test B	n	A+B+	A-B-	A+B-	A-B+
LFA *vs* blood culture	221	15	191	14	1
LFA *vs* LA	224	12	181	18	13
LFA *vs* urine culture	226	11	196	19	0
LFA *vs* CSF culture	65	17	34	10	4
LFA *vs* CSF direct examination	64	15	35	11	3
blood culture *vs* LA	217	8	184	8	17
blood culture *vs* urine culture	217	9	199	7	2
blood culture *vs* CSF culture	64	11	39	5	9
urine culture *vs* CSF culture	65	6	39	5	15

A+B+ = Number of cases positive for both tests
A-B- = Number of cases negative for both tests
A+B- = Number of cases positive for Test A and negative for Test B
A-B+ = Number of cases negative for Test A and positive for Test B

Figure S1

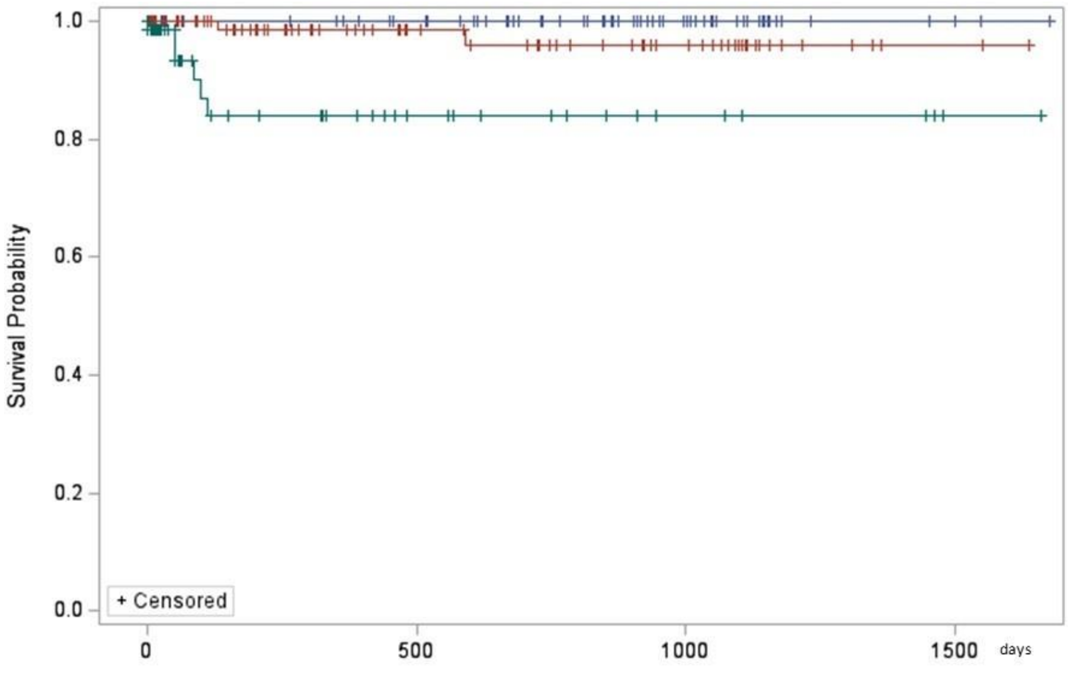

Figure S1. Kaplan-Meier estimator related to place of admission (blue: outpatient; red: Day care hospital; green: admission) (p = 0.0002)

Figure S2

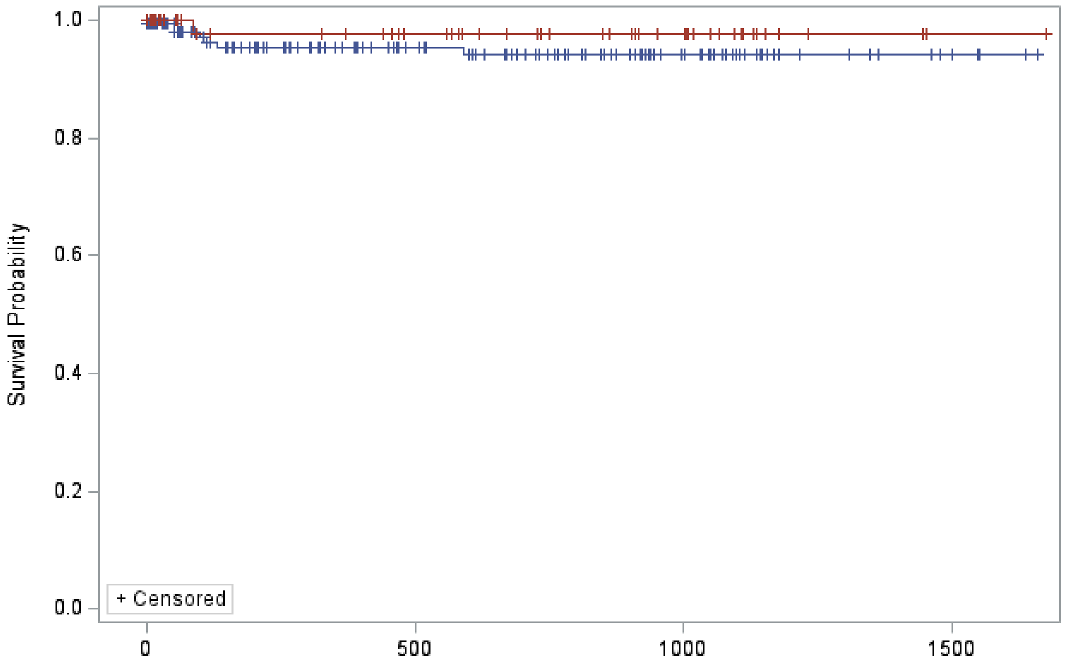

Figure S2. Kaplan-Meier estimator related to sex (blue line- male, red line- female) (p = 0.37).

Figure S3

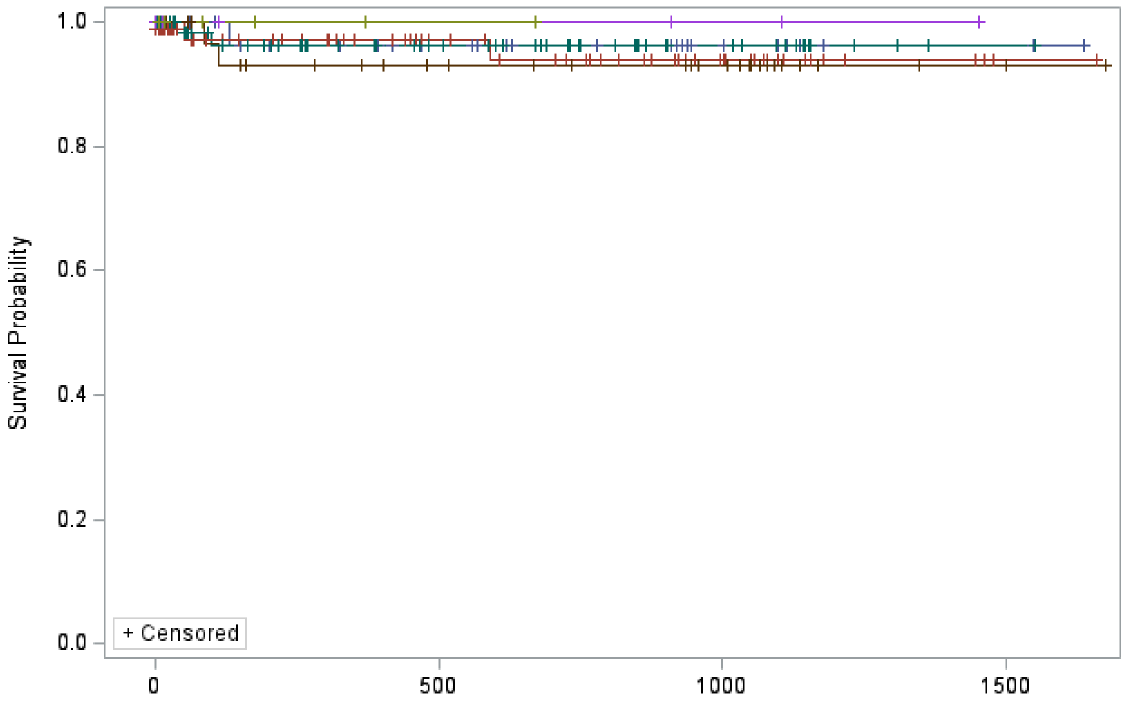

Figure S3. Kaplan-Meier estimator related to age group (blue line: < 29, red line: 30 – 39, green line: 40 – 49, brown line: 50 – 59, purple: 60 – 69, light green: ≥ 70 years old) (p=0.97)

Figure S4

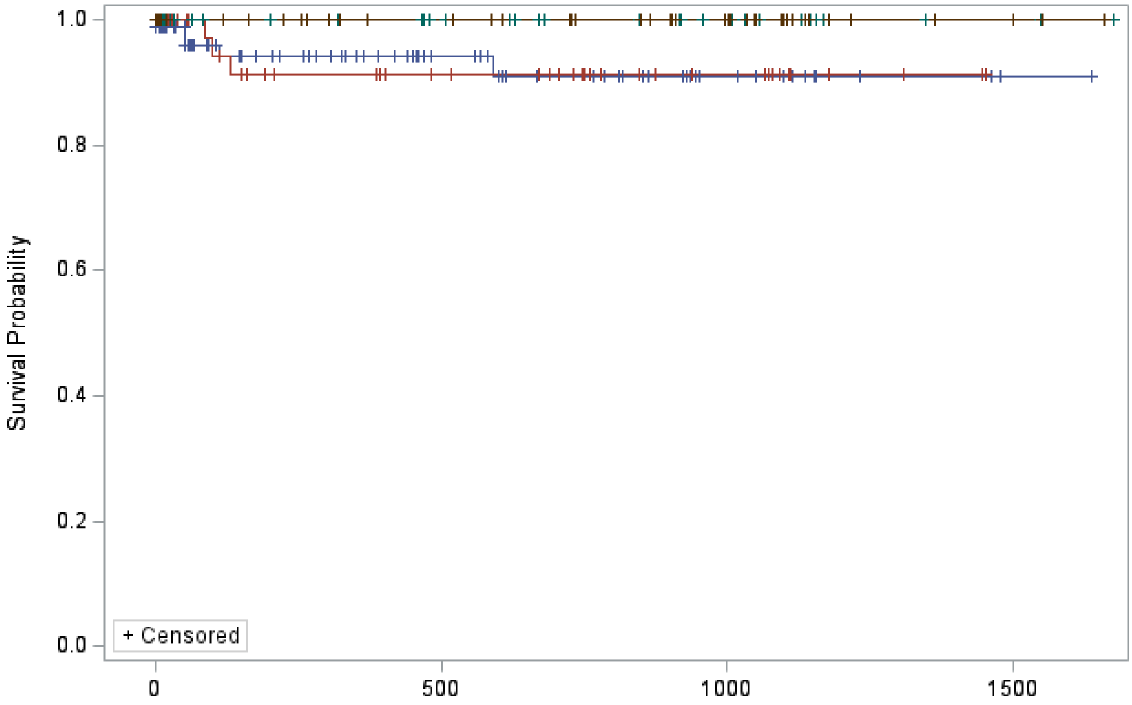

Figure S4. Kaplan-Meier estimator related CD4+ count (blue line: 0 – 50, red line: 51 – 100, green line: 101 – 150, brown line: ≥ 151 cells/mm3) (p = 0.11).
